# Mapping Emerging Scientific Trends in Chronic Skin Disorders Using Machine Learning-Based Bibliometrics

**DOI:** 10.3390/bioengineering12080890

**Published:** 2025-08-20

**Authors:** Nicoleta Cirstea, Andrei-Flavius Radu, Delia Mirela Tit, Ada Radu, Gabriela S. Bungau, Laura Maria Endres, Paul Andrei Negru

**Affiliations:** 1 Doctoral School of Biological and Biomedical Sciences, University of Oradea, 410087 Oradea, Romania; cirstea.nicoleta@student.uoradea.ro (N.C.); dtit@uoradea.ro (D.M.T.); gbungau@uoradea.ro (G.S.B.); lendres@uoradea.ro (L.M.E.); negru.paulandrei@student.uoradea.ro (P.A.N.); 2 Department of Psycho-Neurosciences and Recovery, Faculty of Medicine and Pharmacy, University of Oradea, 410073 Oradea, Romania; 3 Department of Pharmacy, Faculty of Medicine and Pharmacy, University of Oradea, 410028 Oradea, Romania; 4 Department of Preclinical Disciplines, Faculty of Medicine and Pharmacy, University of Oradea, 410073 Oradea, Romania

**Keywords:** scientometrics, skin diseases, Bibliometrix, bibliometric analysis, VOSviewer, Python

## Abstract

Chronic dermatologic diseases are characterized by pathophysiologic complexity and the existence of many unmet patient management needs that can contribute to treatment failure, with poor adherence being a major issue. This study aims to identify key topics in this field, using the Web of Science database. To perform this analysis, tools such as VOSviewer, Bibliometrix, and Excel were used. A Python script leveraging machine learning algorithms was developed to standardize terminology. The initial search yielded 35,373 documents, which were then refined to 12,952 publications spanning 1975 to 2024 through parameter optimization. The study found an increasing interest in this research domain, with a notable surge in 2019. The analysis identified the United States, Germany, and England as the most prolific countries in terms of scientific output. Canada ranked sixth in total document production, but its documents received the highest average citations, reflecting a significant impact. Normalization analysis revealed Italy as the most specialized country in chronic skin disease research relative to total national research output. Trend analysis revealed an evolution in research topics, particularly after 2020, with a growing focus on personalized treatment methods and long-term treatment outcomes. The study highlighted international collaboration, especially among countries with cultural or regional connections, such as those within the European Union. It underscores the growing need for continuous updates and the increasing global focus on chronic skin diseases, highlighting the critical role of staying current with emerging trends to drive advancements in treatment and patient care.

## 1. Introduction

Around 20–25% of individuals are impacted by persistent, non-infectious inflammatory skin conditions [1]. The underlying causes of chronic skin inflammation are diverse and multifactorial [2]. Some of the most common long-term inflammatory skin disorders include atopic dermatitis [3], psoriasis [4], urticaria [5], lichen planus [6], and hidradenitis suppurativa [7], all of which result from a complex interaction between genetic predispositions and environmental influences. Autoimmunity is an additional significant factor contributing to chronic cutaneous inflammation [8].

The complexity of these pathologies at the pathophysiological level has required continuous scientific studies for the development and improvement of existing therapies and, implicitly, for the management of these patients. In the treatment of chronic skin diseases, conventional therapies include topical medications (i.e., steroid creams, emollients), systemic drugs (antibiotics, antihistamines, immunosuppressants), phototherapy, and phytochemicals as adjuvants [9,10]. These are often the first-line options for managing symptoms, controlling inflammation, and itching. On the other hand, biologic therapies represent a more recent approach, using medications that target specific proteins involved in inflammatory processes. These are used in conditions like psoriasis or severe eczema and are typically administered as injections or infusions. Biologic treatment offers more precise disease control, reducing the side effects associated with conventional therapies. The choice of treatment depends on disease severity, previous treatment responses, and potential adverse effects [11].

Evidence-based and consensus-driven guidelines, such as the EuroGuiDerm Guideline and Consensus Statement Development Manual, are essential for the management of these conditions. These resources play a critical role in ensuring accurate and up-to-date therapeutic approaches for the respective pathologies [12,13].

Beyond the foundational biological research that initially guided the exploration of these conditions, there has been a growing focus on assessing the psychological effects, particularly how chronic skin disorders can lead to significant impairment in this domain [14]. Additionally, the evaluation of dermatological research and practice from a sustainable viewpoint has recently captured the attention of the scientific community [15].

Quality of life (QoL) in chronic skin diseases is commonly assessed using specific tools like the Dermatology Life Quality Index (DLQI) and its variants. These instruments evaluate the impact of skin conditions on daily life, including symptoms, emotional well-being, and social interactions. The DLQI is widely used in psoriasis, atopic dermatitis, chronic urticaria, and acne to measure treatment effects and disease burden. For example, the DLQI assesses factors such as itching, pain, and self-esteem [16].

The Global Burden of Disease Studies provide crucial insights into identifying unmet needs that require attention for various conditions, including skin diseases [17,18]. Psoriasis, in particular, shows a rising global prevalence, an increasing burden, and a growing number of associated comorbidities, highlighting the urgent need for interventions to address this significant health disparity worldwide [19].

In light of this concerning situation, it is essential to thoroughly examine all potential factors contributing to treatment failure. At present, inadequate adherence to therapy is a major reason for the ineffectiveness of many treatments. This issue not only significantly impacts the quality of life for patients with chronic conditions but also poses financial challenges for healthcare systems, while further hindering the achievement of favorable clinical outcomes [20]. Therefore, it is crucial to implement and enhance technological tools that support treatment adherence, including teledermatology, gamification, artificial intelligence, web-based patient education, internet surveys, smartphone applications, and text message reminders, as well as to increase the involvement of professionals through high-quality counseling [9,21].

Chronic skin diseases have a significant impact on public health, quality of life, and healthcare costs, yet research on their management and treatment is still developing. Ongoing studies are crucial to refining therapeutic strategies and improving patient outcomes. Adherence to treatment remains a key challenge in managing these conditions. Despite the importance of this issue, bibliometric analyses in this field are sparse and often focus on narrow aspects. This underscores the need for more comprehensive research to better address the complexities of chronic skin diseases and optimize patient care.

The aim of this study was to design a comprehensive bibliometric and science mapping analysis, updating the information on chronic skin diseases. By leveraging modern methodologies, including a custom Python-based thesaurus generator, we have minimized inaccuracies and enhanced the precision of the data. This approach allows for a comprehensive evaluation of research trends, including the identification of the most prolific countries, influential institutions, and key articles within the field. Moreover, the thematic evolution of chronic skin diseases, trending topics, and emerging keywords is highlighted, providing valuable insights into the progression of research in this domain. Bibliometric analysis offers a broad perspective on scientific output, revealing patterns and trends, while also pinpointing gaps in the literature. Understanding these trends is crucial for refining research priorities, optimizing patient care strategies, and advancing the development of targeted therapies. Bibliometric analysis provides practical guidance for researchers in multiple ways. Publication and citation patterns help identify research priorities and funding trends, while institutional and country networks reveal optimal collaboration opportunities. Temporal analysis identifies research gaps requiring attention, highly cited papers establish quality benchmarks and foundational methodologies, and keyword evolution tracking reveals emerging trends and shifting research focus. These indicators transform descriptive statistics into actionable research intelligence for strategic planning and trend identification. This study ultimately aims to contribute to a more accurate, up-to-date understanding of the field’s evolution.

## 2. Materials and Methods

**Figure 1:**
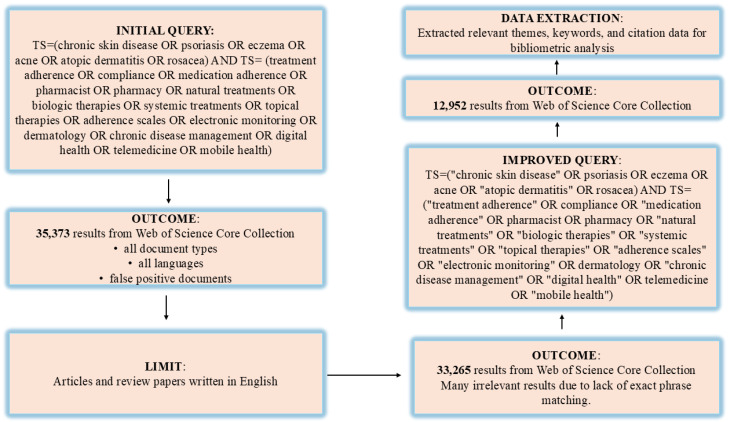
Methodology for document identification and selection in bibliometric research.

In conducting this research, bibliometric data from the Web of Science (WOS) database [22] were subject to analysis. The process of data collection entailed a comprehensive search employing well-defined keywords tailored to the subject matter under investigation. Figure 1 illustrates the methodological scheme employed, delineating the stages of query development, application of selection criteria, and progressive refinement of results. The initial search yielded 35,373 documents, which were systematically refined through sequential filtering stages to ensure precision and relevance. Document type restriction to articles and reviews eliminated conference papers, editorials, and other non-peer-reviewed materials (stage 1). Language filtering retained only English-language publications to ensure consistent analysis (stage 2). Implementation of exact phrase matching using quotation marks significantly reduced false positives by requiring precise terminology matches (stage 3). This systematic approach yielded 12,952 publications representing all documents that met our comprehensive inclusion criteria, with no arbitrary numerical cutoffs applied. These modifications resulted in a significant decrease in false positive outcomes. The employed algorithm was pivotal in assembling a concentrated and precise dataset for bibliometric examination. The chosen documents were exported in tab-delimited format via the “Export” functionality accessible through WOS, selecting the “Full Record and Cited References” option to encompass all pertinent data.

The analysis employed bibliometric methods utilizing VOSviewer (version 1.6.20, Centre for Science and Technology Studies, Leiden University, Leiden, The Netherlands), Bibliometrix (Università degli Studi di Napoli Federico II and University of Campania Luigi Vanvitelli, Naples, Italy) [23] via the Biblioshiny web platform, and Microsoft Excel (Microsoft Corporation, Redmond, WA, USA). These software tools were employed to examine scientific data extracted from databases such as WOS and Scopus, thus enabling the exploration of trends, collaborative relationships, and the progression of research themes [24,25]. Data processing and visualization were performed using Python 3.12.3 (Python Software Foundation, Wilmington, DE, USA) [26] with the following libraries: Pandas 2.3.1 for data manipulation and statistical calculations [27], NumPy 2.3.2 for numerical operations [28], Matplotlib 3.10.5 for creating figures and charts [29].

For normalization analysis, a parallel search was conducted using the topic search TS = (“dermatology”) to establish baseline dermatology publication volumes for the same temporal period (1975–2024), enabling calculation of the proportional representation of chronic skin disease research within the broader dermatological literature.

To assess country-specific research specialization, total publication volumes for each country were obtained through Web of Science address field searches (CU = [country name]) encompassing articles and reviews for the same temporal period, enabling calculation of specialization indices representing the proportion of each country’s total research output dedicated to chronic skin disease studies.

Institutional research specialization was assessed by obtaining total publication volumes for leading institutions through Web of Science affiliation searches, enabling calculation of specialization indices representing the proportion of each institution’s research output dedicated to chronic skin disease studies, thereby distinguishing genuine institutional expertise from general research capacity.

Temporal institutional specialization trends were analyzed by obtaining yearly publication volumes for leading institutions, enabling calculation of annual specialization indices to distinguish authentic research focus evolution from proportional expansion with general institutional research capacity over the study period (1991–2024).

The number of papers published by each country is indicated by the size of the node in the country co-authorship network map; the larger the bubble, the more papers that country has contributed. The degree of collaboration between two countries is indicated by the thickness of the connecting lines; the thicker the line, the closer the two countries work together. Based on the color of the bubbles and lines, countries are organized into clusters that show which countries work together the most frequently. Countries in the same cluster tend to have stronger working partnerships.

In the journal collaboration network map, each node represents a publication source, with its size reflecting the number of papers published by that source. The color of each node indicates the average publication year of the source’s papers, offering insights into temporal publishing trends. Connections between nodes represent citations, where one source references another. The thickness of these connections signifies the strength of the citation relationship, with thicker lines denoting a higher frequency of citation exchanges between the two sources.

Thematic evolution maps provide a visual representation of the evolution of research topics over time, highlighting changes in focus and the rise or decline of specific topics within a field. Created in Bibliometrix using co-word analysis and clustering techniques, these maps provide valuable insights into the changing dynamics of scientific knowledge over different time periods.

**Figure 2:**
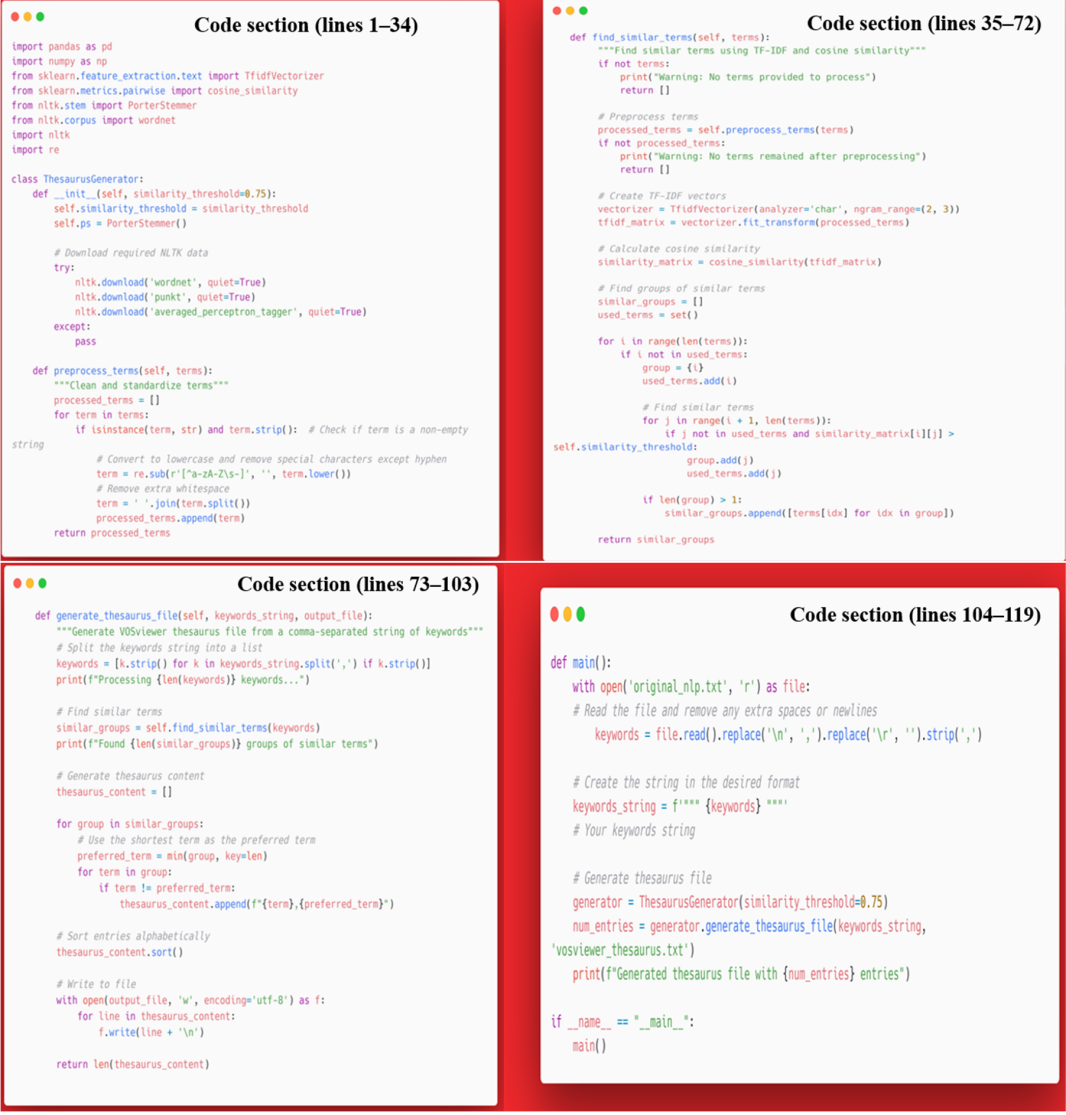
Custom Python-based thesaurus generator.

A prerequisite for conducting a bibliometric analysis that yields satisfactory results is the normalization of the terms under scrutiny. This process is imperative as the same terms may be expressed in a variety of forms. It is also of paramount importance to normalize the nomenclature of countries, as they may be found in different variants (e.g., Turkiye and Turkey). For the analysis of countries, manual normalization is feasible due to the relatively low number of countries compared to the number of keywords analyzed in a bibliometric study (more than 23,000 in this analysis). To handle the large volume of data, a machine learning-based approach was implemented using Python [30]. A custom Python-based thesaurus generator (Figure 2, 119 lines of code and Supplementary File S1) was developed to improve the analysis of keyword trends and thematic evolution in bibliometric studies. This tool leverages various techniques, such as TF-IDF vectorization, cosine similarity, and text preprocessing, to analyze the keyword list and group terms that are semantically or lexically similar. The algorithm selects the shortest term as the preferred label for each group and generates a thesaurus file compatible with VOSviewer. To validate the accuracy of our automated keyword processing, we randomly selected 300 keyword label pairs from the thesaurus output for manual verification. Three researchers from our team independently reviewed each pair to confirm correct term clustering and preferred label selection. The validation achieved at least 90% accuracy. The automated algorithm was refined based on disagreement cases until reaching this minimum 90% accuracy threshold, ensuring reliable processing of our large keyword dataset. This method enhances keyword standardization by consolidating synonyms and variations, thereby improving the accuracy and clarity of bibliometric visualizations.

## 3. Results

### 3.1. Literature Overview

**Figure 3:**
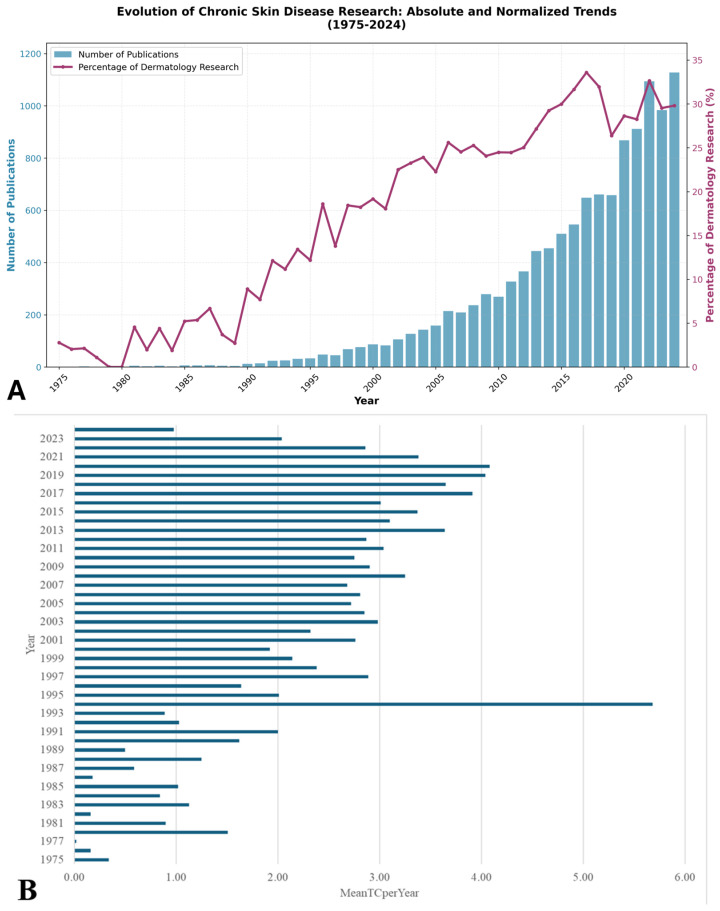
(**A**): Annual evolution of publications targeting chronic skin disease management between 1975 and 2024, absolute publication numbers (blue bars, left axis) and normalized percentage of total dermatology research (red line, right axis); (**B**): MeanTCperYear parameter.

The analysis reveals that, between 1975 and 2025, a total of 11,945 publications on adherence to treatments for chronic skin conditions were published, with a significant trend of increasing interest in this area being demonstrated. There was a gradual increase in the number of publications from 1975 to 2010. Thereafter, a more rapid increase was observed, culminating in a notable increase in 2020, when the number of publications rose from 658 in 2019 to 868. Figure 3A illustrates the annual evolution of publications between 1975 and 2024, while Figure 3B shows the MeanTCperYear parameter. This metric, which reflects the average number of citations an article receives annually, is calculated by dividing the total number of citations by the number of years since publication.

To contextualize this publication growth within the broader dermatological research landscape, we normalized our findings against total dermatology publications retrieved using topic search methodology. The analysis reveals that chronic skin disease research represented 2.8% of all dermatology publications in 1975, increasing substantially to 29.8% in 2024, demonstrating a 10.7-fold increase in relative research attention. This growth pattern significantly exceeds proportional expansion, with the field achieving peak representation of 33.6% in 2017. The temporal analysis shows distinct phases: the early period (1975–1995) averaged 5.2% of dermatology research, while the recent period (2010–2024) maintained an average of 28.9%, confirming a genuine increased research focus rather than general publishing expansion.

### 3.2. Country Scientific Production

According to the data analyzed using VOSviewer, a total of 121 countries contributed to the scientific output on adherence to treatments for chronic skin conditions during this period. Among these, only 33 countries produced more than 100 articles on the subject. The United States stands out as the most prolific country, with 3615 documents, followed by Germany with 1237 documents and England with 1136 documents. Table 1 highlights the top 10 countries in terms of productivity in this research area. In terms of average citations per document, Canada excels with an impressive average of 43.22 citations per document, despite having produced fewer documents (679) compared to other highly productive countries. Similarly, France and England demonstrate notable impact with averages of 41.83 and 41.59 citations per document, respectively.

Normalization against total national research output reveals distinct patterns of research specialization beyond absolute productivity metrics. Italy demonstrates the highest research specialization index (0.047%), indicating nearly five publications per 10,000 total research outputs focus on chronic skin diseases, followed by Germany (0.036%) and England (0.029%). Despite lower absolute publication volumes, this analysis identifies countries with proportionally greater research commitment to the field. Notably, China shows the lowest specialization index (0.009%) despite substantial total research capacity, while Canada and France maintain moderate specialization levels (0.028% and 0.021%, respectively) alongside high citation impact per document.

**Figure 4:**
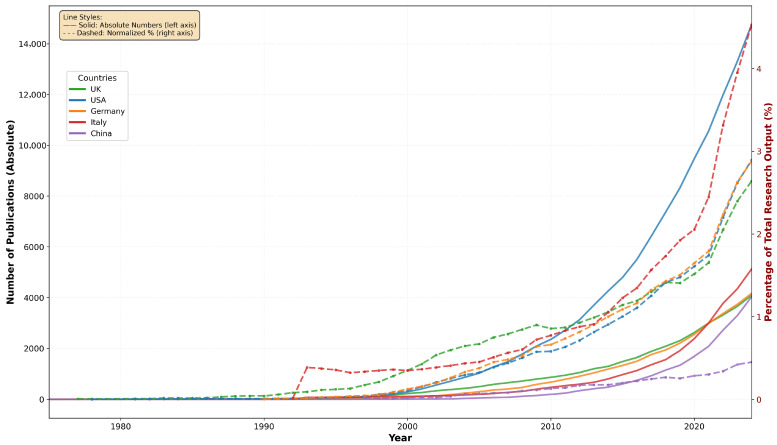
Country publication trends in chronic skin disease research: absolute numbers (solid lines, left axis) and normalized percentage of total national research output (dashed lines, right axis), 1975–2024.

Temporal analysis of country-specific research output reveals distinct patterns when examining both absolute productivity and normalized research focus (Figure 4). The United States demonstrates the most substantial absolute growth trajectory, increasing from minimal publications in the 1970s to over 14,000 chronic skin disease publications by 2024. Germany and the United Kingdom exhibit parallel growth patterns with sustained research output expansion throughout the study period. However, normalization against total national research output provides crucial insights into genuine research specialization trends.

The normalized analysis reveals remarkable temporal evolution in research specialization across all leading countries. Italy achieves the highest research focus, with chronic skin disease studies representing 3.26% of total national research output in recent years (2020–2024), peaking at 4.53% in 2024. Germany (2.24% recent average, 2.88% peak) and the USA (2.21% recent average, 2.89% peak) demonstrate similarly high specialization levels, while the United Kingdom maintains substantial focus at 2.05% average with 2.64% peak specialization. Comparison with earlier periods (1995–2005) reveals dramatic increases in research attention: Germany increased from 0.16% to 2.24%, the USA from 0.14% to 2.21%, and Italy from 0.38% to 3.26%. China presents contrasting patterns, with recent specialization of 0.36% representing substantial growth from early levels (0.03%), yet remaining the lowest among leading countries. These temporal normalization patterns definitively confirm that observed growth trends reflect genuine increased research interest and national specialization rather than general scientific productivity expansion.

### 3.3. Institution Scientific Production

**Figure 5:**
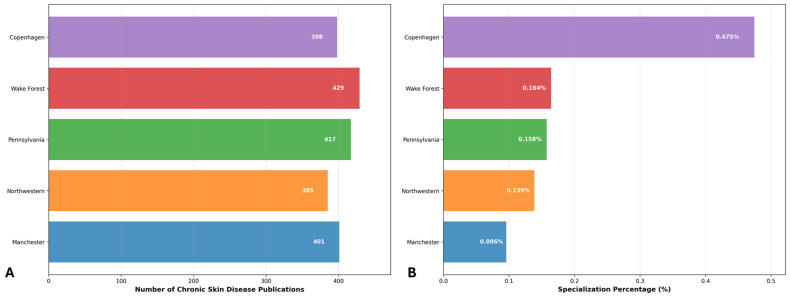
Institutional research profile in chronic skin disease studies: (**A**) absolute publication output and (**B**) specialization percentage of total institutional research, highlighting the distinction between research volume and specialized focus (1975–2024).

Institutional analysis reveals distinct patterns when examining both absolute research productivity and normalized specialization focus (Figure 5). The University of Manchester demonstrates the highest absolute output with 429 chronic skin disease publications, followed by Northwestern University (417 publications) and the University of Pennsylvania (401 publications). However, normalization against total institutional research output provides crucial insights into genuine research specialization.

Wake Forest University exhibits the most remarkable specialization profile, dedicating 0.475% of its total research output to chronic skin disease studies, representing 4.9-fold higher specialization than the least focused institution. Despite ranking fourth in absolute productivity (398 publications), Wake Forest’s specialization index explains its historical research leadership documented in temporal analysis. The University of Manchester, while leading in absolute output, maintains substantial specialization (0.164%) alongside Northwestern University (0.158%). The University of Copenhagen demonstrates moderate specialization (0.139%) with consistent research contribution. Notably, the University of Pennsylvania, despite possessing the largest total research capacity (418,220 publications), exhibits the lowest specialization index (0.096%), indicating that chronic skin disease research represents a minimal proportion of its extensive research portfolio. These findings confirm that institutional leadership in chronic skin disease research reflects genuine specialized commitment rather than general research volume, with smaller, focused institutions often demonstrating proportionally greater expertise than larger research centers.

A thorough review of the scientific production data generated by Bibliometrix over time (affiliation data available since 1991) reveals a consistent upward trend across all institutions in the dataset except Wake Forest University, which has maintained its position at the top of the rankings until 2020, when it was surpassed by the University of Manchester. The early period (1991–2000) was characterized by Wake Forest University’s marked dominance in research output, particularly following a substantial increase in publications between 1994 and 1996. During this initial phase, other institutions maintained relatively modest publication rates, typically below ten publications annually. This period established the baseline for subsequent institutional development in research productivity. The intermediate period (2000–2015) witnessed a transformation in the research landscape. While Wake Forest University continued to lead in absolute numbers, other institutions began demonstrating remarkable growth trajectories. The University of Pennsylvania initiated a significant expansion in research output post-2005, while the University of Manchester and the University of Copenhagen exhibited parallel growth patterns, suggesting similar institutional developments in research capacity and output. The most recent period (2015–2024) has been characterized by a fundamental restructuring of the relative positions among these institutions, with a notable shift occurring around 2020 when the University of Manchester superseded Wake Forest University’s long-standing leadership position. Northwestern University demonstrated particularly impressive growth during this period, with a marked acceleration in publication output. By 2024, the data indicates a convergence in research productivity across all five institutions, with annual publication numbers ranging from 385 to 429.

**Figure 6:**
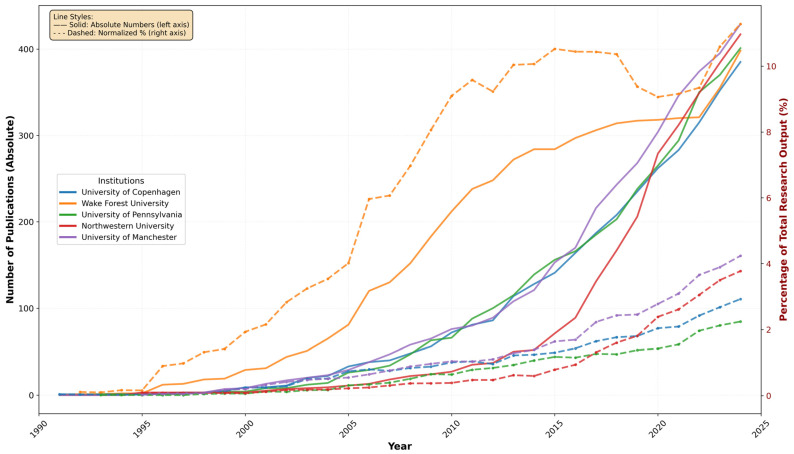
Temporal institutional publication trends in chronic skin disease research: absolute numbers (solid lines, left axis) and normalized percentage of total institutional research output (dashed lines, right axis), demonstrating genuine specialization evolution independent of general research capacity expansion (1991–2024).

Temporal normalization analysis reveals compelling evidence of genuine institutional research specialization evolution beyond general productivity expansion (Figure 6). All leading institutions demonstrate substantial increases in research focus over time, with temporal correlations ranging from 0.853 to 0.961, confirming consistent specialization growth. Wake Forest University exhibits the most dramatic pattern, increasing from 2.04% average specialization (1995–2005) to 9.89% (2020–2024), achieving peak specialization of 11.28% in 2024. The University of Manchester demonstrates exceptional evolution, growing from 0.26% early-period specialization to 3.53% recent average. The University of Pennsylvania shows steady specialization growth from 0.10% to 1.86% despite its substantial institutional size, while Northwestern University and the University of Copenhagen maintain consistent upward trajectories. These temporal patterns definitively confirm that observed institutional growth represents authentic increased research commitment to chronic skin disease management rather than proportional expansion with general research infrastructure, with all institutions achieving peak specialization levels in 2024.

### 3.4. Most Influential Papers in Adherence Research for Chronic Dermatologic Conditions

An analysis of the thematic focus of the most influential publications in adherence research for chronic dermatological conditions shows that the majority of these papers focus on the management of dermatological problems. The most influential article from this period deals with the DLQI, a widely used tool for categorizing the impact of dermatological conditions on patients’ quality of life. Psoriasis is another topic that has received considerable attention. Key articles by Paris R, Leonardi CL, and Armstrong AW focus on this condition. In particular, this topic has shown a sustained interest over the period under review (1975–2024), as evidenced by the continuous publication of articles on this topic, which is evident from the bibliometric analysis. Table 2 summarizes the top 10 most influential papers in the field of adherence research for chronic dermatological conditions from 1975 to 2024.

### 3.5. Visualizing Scientific Networks and Trends

#### 3.5.1. Country Collaborations in the Evaluated Field

**Figure 7:**
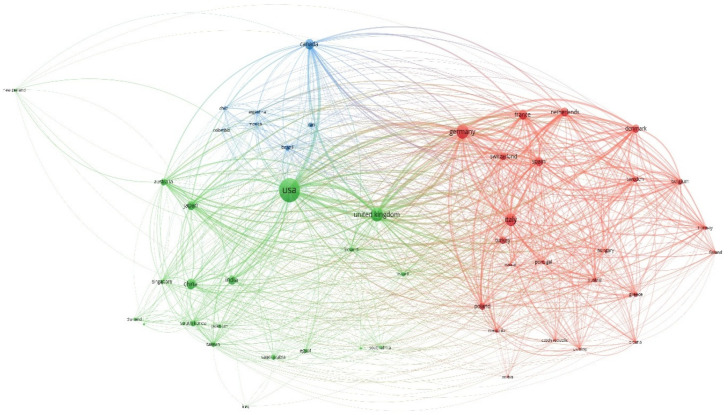
Map of country collaborative networks for adherence studies in chronic dermatological conditions (1975–2024).

Analysis of the cross-country collaboration map generated by VOSviewer identified three distinct clusters of collaboration (Figure 7). The green cluster is led by the United States, the most prolific country in this area. It is surrounded by countries from the Asia-Pacific region, indicating strong collaboration between these nations. The red cluster is led by Germany and includes mainly European countries. This suggests that intra-European collaboration programs are fostering successful interactions within this region. The blue cluster is led by Canada and is closely linked to the green cluster, particularly the United States, highlighting a strong partnership between the United States and the countries in this cluster.

#### 3.5.2. Thematic Evolution in the Field of Adherence to Treatments for Chronic Skin Diseases

**Figure 8:**
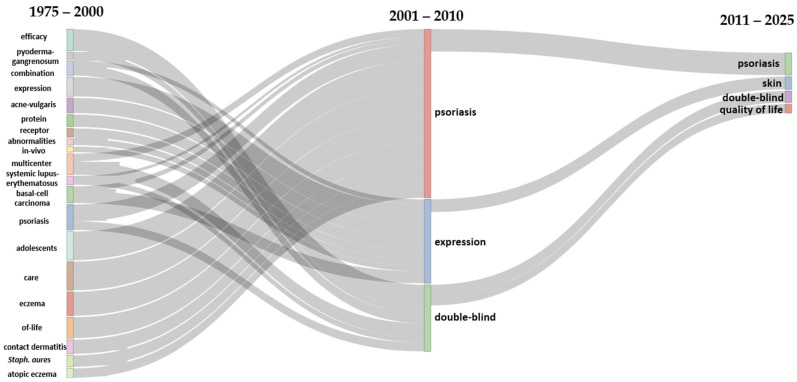
Thematic evolution in the field of adherence to treatments for chronic skin diseases (1975–2000, 2001–2010, 2011–2025).

From 1975 to 2000, studies on adherence to treatments for chronic skin diseases were predominantly focused on foundational research endeavors that sought to comprehend treatment effectiveness and biological mechanisms. During this period, significant attention was directed towards specific conditions such as psoriasis, systemic lupus erythematosus, and atopic eczema. Additionally, broader subjects, encompassing in vivo studies and receptor-related research, attained prominence. Innovative methodologies, including multicentre trials, were frequently employed, showcasing the era’s dedication to establishing a robust scientific foundation. Furthermore, research began to explore patient demographics, with adolescents and aspects of care management emerging as notable areas of focus. Between 2001 and 2010, the research focus evolved to emphasize the consolidation of knowledge through more robust clinical studies. Psoriasis emerged as a key focal point, representing its growing significance as a model for exploring adherence to treatments, and the widespread adoption of “double-blind” trials during this time highlighted the increasing reliance on evidence-based approaches. Concurrently, studies on gene and protein expression continued to deepen understanding of the biological processes underlying chronic skin conditions, and this phase marked a transition from exploratory efforts to more systematic and clinically validated research methodologies. From 2011 onwards, a notable shift occurred, with the focus shifting towards patient-centered approaches and the concept of “quality of life” becoming a prominent theme. While psoriasis remained a central topic, research expanded to encompass a broader range of dermatological health issues, as reflected by the emphasis on “skin” as a recurring subject. The sustained use of double-blind trials underscores the ongoing prioritization of clinical rigor in evaluating adherence strategies. This period also reflects a more comprehensive approach to managing chronic skin diseases, focusing on both treatment efficacy and the overall well-being of patients. The thematic evolution map is depicted in Figure 8.

#### 3.5.3. Trending Evolution in the Field of Adherence to Treatments for Chronic Skin Diseases

The alterations in the prevalence of trending topics over time serve as an indicator of the manner in which researchers engage with and investigate chronic skin diseases. The analysis conducted reveals more than merely divergent research domains; it demonstrates the evolution of scientific language and the way researchers disseminate their findings. When related terms such as ‘psoriasis’, ‘dermatology’, and ‘dermatitis’ emerge as separate trends during analogous time periods, this does not imply that researchers are studying wholly distinct subjects. Instead, it demonstrates that the field is developing more sophisticated ways to describe and discuss the same research areas.

**Figure 9:**
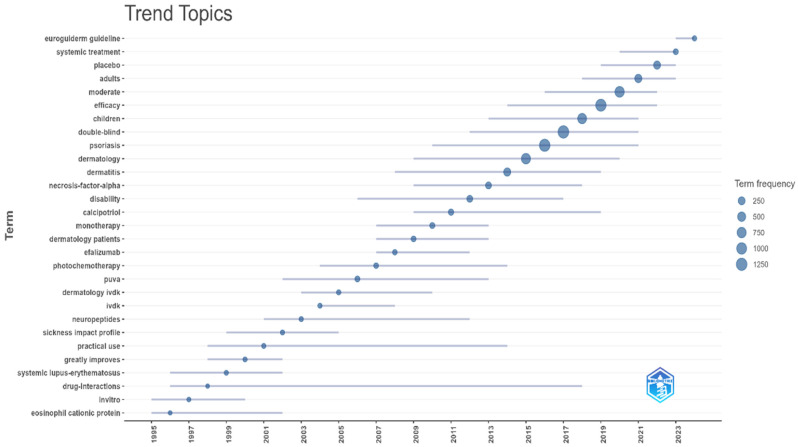
Trend topics in the field of adherence to treatments for chronic skin diseases.

The way research topics have changed over time for treatments for chronic skin diseases shows different phases of focus, driven by both clinical and societal needs. Figure 9 highlights the trending topics based on the year. These evolving trends underscore the dynamic nature of adherence research in chronic skin diseases, highlighting the field’s response to both scientific advancements and changing societal needs. From 2011 onwards, research has taken a more holistic and patient-centered approach. Themes such as quality of life, disability, and studies on specific demographics like children and adults emphasize the importance of understanding the broader implications of treatment adherence on patients’ well-being. Meanwhile, the rise in topics like systemic treatments and EuroGuiDerm guidelines reflects a growing commitment to evidence-based, standardized approaches to care.

#### 3.5.4. Trending Topics in the Field of Adherence to Treatments for Chronic Skin Diseases

**Figure 10:**
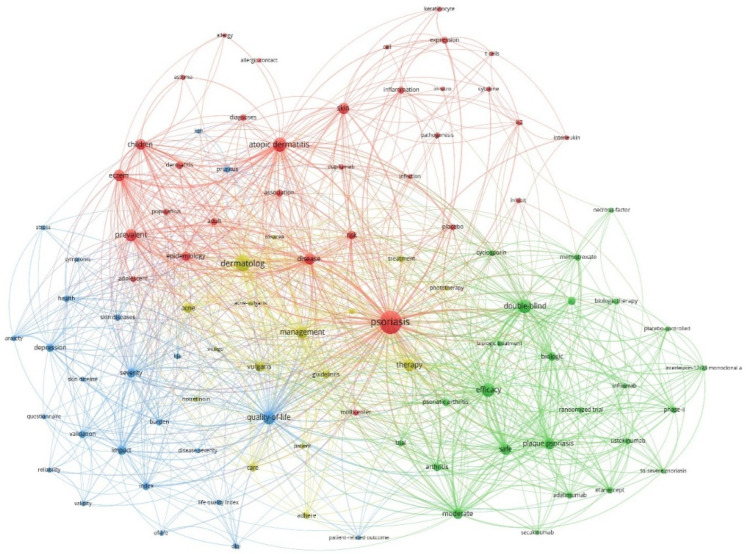
Keyword co-occurrence network in the field of adherence to treatments for chronic skin diseases (1975–2024).

Based on the visualization analysis conducted through VOSviewer, the network map reveals four distinct research clusters, featuring keywords that meet a minimum threshold of 150 occurrences (Figure 10). Each cluster represents a specialized domain within the field of waste management, highlighting different research priorities and focus areas. The largest group, the red one, contains 25 keywords and is mostly about atopic dermatitis and how it works. This is shown by keywords such as inflammation and pathogenesis, which emphasize the clinical aspects of atopic dermatitis. The second-largest cluster (the green one) focuses on clinical trials and treatments, with keywords like double-blind, placebo-controlled, and biological therapy. The blue cluster is centered on assessing patients and outcomes, represented by keywords like quality of life, severity, validation, and reliability. The smallest group, the yellow one, is about how to manage the condition and includes 16 keywords.

#### 3.5.5. Emerging and Well-Established Journals in the Field of Adherence to Treatments for Chronic Skin Diseases

**Figure 11:**
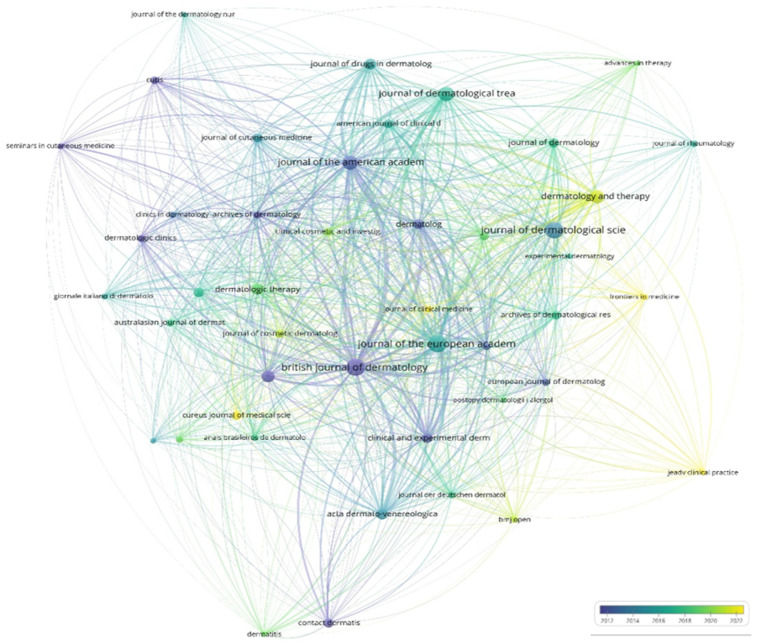
Citation network map showcasing both established and emerging trends in the field of adherence to treatments for chronic skin diseases (1975–2024).

The network map of citations (Figure 11) shows that the British Journal of Dermatology and the Journal of the European Academy are very important in dermatological research. They are in the middle of the network and have a lot of connections, which shows that they have a lot of influence in this field. If we look at the color gradient timeline (2012–2022), we can see how publication patterns have changed over time. Journals in blue, like the British Journal of Dermatology, show earlier publications (around 2012–2014) and are a sign of their long-standing presence and contribution to the field. Journals in yellow/green, like Frontiers in Medicine and Advanced Therapy, are more towards the edge of the network and are more recent publications (around 2020–2022). This suggests that there are new areas of interest and new places where dermatological research is being published.

## 4. Discussion

The temporal evolution of research topics in adherence to treatments for chronic skin diseases highlights distinct phases of focus, driven by both clinical and societal needs. Early research from the 1990s to the early 2000s concentrated on foundational biological studies and clinical exploration, with topics such as eosinophil cationic protein, systemic lupus erythematosus, and drug interactions reflecting an emphasis on understanding disease mechanisms, pharmacological complexities, and baseline clinical efficacy. This foundational research established the foundation for more targeted interventions. Between 2001 and 2010, there was a shift towards the development and evaluation of new treatments and methodologies, with topics such as PUVA and photochemotherapy emerging, showcasing advancements in therapeutic options. Concurrently, the prominence of double-blind trials highlights an era of methodological rigor aimed at validating treatment adherence strategies.

Conditions such as psoriasis garnered heightened attention, thereby consolidating their status as model diseases for the exploration of adherence challenges in chronic dermatological conditions. Concurrently, the rise in topics such as systemic treatments and EuroGuiDerm guidelines signifies a growing commitment to evidence-based, standardized approaches to care. A notable recent trend, especially from 2020 onward, is the integration of sustainability into dermatological research and practice. Terminology such as placebo and moderate point to nuanced, precision-based approaches in clinical trials, while the emergence of systemic treatments and updated guidelines suggests an alignment with global shifts towards more comprehensive and sustainable healthcare practices.

The substantial growth in chronic skin disease research demonstrated through our bibliometric analysis represents genuine increased scientific interest rather than artifact of general publishing expansion. Normalization against total dermatology publications reveals that this research domain increased from 2.8% of dermatological studies in 1975 to 29.8% in 2024, constituting a significant increase in relative research attention. This proportional growth significantly exceeds baseline dermatology publishing trends, with the field achieving sustained representation averaging 28.9% of dermatology research since 2010 compared to 5.2% in the foundational period (1975–1995). The peak research focus of 33.6% in 2017 underscores the field’s emergence as a dominant area within dermatological sciences, reflecting both clinical urgency and therapeutic complexity inherent in chronic skin disease management.

Country-level analysis reveals that research leadership extends beyond absolute publication volumes to include specialization patterns. Italy’s highest specialization index (0.047%) suggests a strategic national research focus, while Germany’s combination of high productivity (1237 documents) and specialization (0.036%) indicates sustained institutional commitment to the field.

The institutional normalization analysis reinforces the principle that research excellence extends beyond absolute productivity to encompass specialized focus. Wake Forest University’s exceptional specialization index (0.475%) provides a compelling explanation for its sustained historical leadership in chronic skin disease research, demonstrating that smaller institutions with targeted expertise can achieve disproportionate impact compared to larger research centers. This finding underscores the importance of institutional commitment and specialized research programs in advancing specific scientific domains, rather than relying solely on general research infrastructure capacity.

The collaboration network reveals a clear pattern of interactions between countries, with distinct clusters representing regions or groups of countries with strong research and academic partnerships. These clusters are likely to result from shared research interests, geographic proximity, historical ties, or participation in multinational research programs and initiatives. For instance, the red cluster, which is primarily made up of European countries, indicates a robust network of collaboration, which can be attributed to frameworks such as the European Union’s Horizon Europe program, which encourages cross-border cooperation in science and innovation. These countries frequently share analogous objectives, funding mechanisms, and access to shared research infrastructures, thereby facilitating the establishment of long-term partnerships. In contrast, the green cluster emphasizes collaborations among countries in Asia, Oceania, and North America, suggesting a distinct dynamic, potentially driven by economic partnerships, technology transfer agreements, or participation in global challenges such as climate change and public health. The blue cluster, comprising countries such as the USA and Canada, underscores the pivotal role of global hubs in facilitating widespread international connections, acting as intermediaries between multiple regions. These clusters offer invaluable insights into how geopolitical, economic, and scientific factors influence global research networks. By comprehending these connections, policymakers and researchers can identify potential areas for enhancing collaboration or addressing disparities in international research endeavors.

In the early 1990s, the focus of dermatology research was primarily on the establishment of fundamental measurement tools and the comprehension of fundamental disease impacts. The seminal development of the DLQI by Finlay and Khan in 1994 [31] represented a pivotal moment, introducing the first standardized methodology for the measurement of the impact of skin conditions on patients’ lives. Concurrently, researchers initiated exploration of the psychological dimensions of skin diseases, acknowledging that the impact transcended physical symptoms. As the late 1990s and early 2000s approached, research underwent a substantial expansion with a view to gaining a more comprehensive understanding of specific conditions and their broader effects. A remarkable study in this regard was that of Gupta and Gupta (1998) [32], which examined depression and suicidal ideation in dermatology patients. This research highlighted the significant psychological impact of skin conditions. Concurrently, the emergence of more sophisticated treatment approaches became evident, particularly with the introduction of biological therapies and the development of targeted treatments for conditions such as psoriasis.

The 2000s brought a remarkable shift toward evidence-based medicine and standardized treatment protocols. Researchers began conducting larger-scale studies to establish treatment efficacy and safety profiles. The field also saw significant advancement in therapeutic options, with studies exploring photodynamic therapy and other innovative treatment approaches. This decade was characterized by a growing recognition of the need to balance clinical efficacy with patient experience and quality of life. The 2010s marked a transformation toward more comprehensive and global perspectives. Major epidemiological studies, such as the Global Burden of Disease Study in 2010 [33], provided unprecedented insights into the worldwide impact of skin conditions. This period also saw the development of more sophisticated biological treatments, particularly for psoriasis, with drugs targeting specific molecular pathways. The emergence of patient-centered outcome measures and treatment guidelines reflected a more holistic approach to dermatological care. In recent years (2020 onwards), research has increasingly focused on personalized medicine approaches and long-term treatment outcomes. Studies have highlighted the importance of tailoring treatment strategies to individual patient factors and preferences. There has also been an increased focus on the economic burden of skin disease and the need for cost-effective treatment solutions.

This evolution reflects a fundamental shift in dermatology research from a purely clinical focus to a more comprehensive understanding of skin disease, encompassing physical, psychological, social, and economic dimensions. The field has moved from basic measurement tools to sophisticated assessment methods, from generic treatments to targeted therapies, and from localized studies to global perspectives. This progress has led to more effective, patient-centered approaches to the management of dermatological conditions, although challenges remain in ensuring accessible and effective treatment for all patients.

The trends suggest that future research is likely to continue to focus on personalized medicine approaches, long-term disease management strategies, and the development of more targeted therapeutic options, while maintaining an emphasis on patient quality of life and accessibility to care.

Based on our comprehensive analysis, several strategic recommendations emerge for advancing chronic skin disease research and practice. Researchers should prioritize collaboration with institutions in Italy and Germany, which demonstrate the highest specialization indices (0.047% and 0.036%, respectively), while leveraging Wake Forest University’s model of achieving disproportionate impact through focused expertise rather than general research volume. Our trending analysis reveals critical research gaps in personalized medicine approaches and long-term treatment adherence strategies that warrant immediate attention. Funding agencies should recognize chronic skin disease research as a priority domain, given its growth from 2.8% to 29.8% of dermatological research, supporting interdisciplinary initiatives that combine clinical efficacy with quality-of-life outcomes. Clinical practitioners should embrace the patient-centered approaches highlighted in our post-2011 thematic evolution, implementing evidence-based guidelines such as EuroGuiDerm while focusing on sustainable long-term management strategies. International collaborative networks should be strategically leveraged, particularly the European research cluster and Asia-Pacific partnerships identified in our analysis, to accelerate knowledge transfer and therapeutic innovation. These recommendations provide actionable pathways for translating our bibliometric findings into tangible advances in chronic skin disease management and patient outcomes.

Bibliometric analyses are invaluable for uncovering patterns and trends in large areas of research. A key advantage is their ability to process large datasets, providing a broad perspective on scientific output. However, this strength can also pose challenges. The sheer volume of articles makes it impractical to manually validate each one, potentially leading to the inclusion of inaccurate or irrelevant entries. Despite rigorous filtering procedures, the large volume of articles (12,952) prevents individual manual verification of each study’s thematic alignment. While our systematic filtering approach using exact phrase matching and content validation was designed to ensure relevance, some articles might be tangentially related to but not directly focused on chronic skin disease treatment adherence.

Another limitation is language bias. Studies published in languages other than English are often excluded, which may inadvertently omit important findings and limit the scope of the analysis. In addition, citation bias can distort results. Highly cited papers do not necessarily represent the most innovative research, especially if they are published in niche journals with a limited readership. In addition, practices such as excessive self-citation or coordinated citation exchanges between collaborators or editorial teams can artificially inflate citation metrics, distorting the perceived impact of research.

A further limitation concerns potential keyword interdependence in thematic evolution analysis. Keywords such as ‘skin’ and ‘psoriasis’ may represent overlapping rather than independent research domains, with condition-specific studies frequently employing broader dermatological terminology. Our thematic evolution analysis reveals apparent shifts from condition-specific to general terminology over time, but this could reflect either genuine research diversification or linguistic evolution within concentrated research areas rather than true thematic independence.

Despite these limitations, bibliometric methods remain an indispensable tool for researchers, academics, and students. They provide an efficient way to identify patterns in scientific output and to evaluate academic performance. Ongoing advances in machine learning and artificial intelligence are expected to address many existing challenges, such as language barriers, data inaccuracies, and citation biases. These technological improvements promise to refine the precision and depth of bibliometric studies, allowing for more accurate assessments of research quality and emerging trends. As a result, the benefits of these analyses will become even more significant. However, it is important to approach their findings with an understanding of their inherent limitations in order to ensure a balanced interpretation.

## 5. Conclusions and Prospects

The bibliometric evaluation of the dermatological treatment adherence literature illuminates the dynamic evolution of this research field. Over the past three decades, scholarly focus has undergone a remarkable transformation, moving from fundamental laboratory investigations to increasingly sophisticated therapeutic approaches. The analysis reveals distinct patterns of international scientific collaboration, with particularly robust research networks emerging across Europe, the Asia-Pacific region, and across North America, demonstrating the truly international scope of contemporary dermatological research.

The field’s progression can be traced through several key developments. The early 1990s saw groundbreaking work in establishing foundational assessment tools, notably exemplified by the creation of standardized quality-of-life measurements. As understanding deepened, research expanded to encompass the multifaceted nature of skin conditions, acknowledging both their physiological and psychological dimensions. Contemporary studies, particularly those from 2020 onward, have increasingly emphasized precision medicine, sustainable healthcare practices, and evidence-based treatment protocols.

The trajectory of dermatological research points toward several promising directions. The integration of advanced technologies, personalized therapeutic approaches, and comprehensive patient care models suggests a future where treatment strategies are increasingly tailored to individual needs. While bibliometric methodologies face certain constraints, including potential language and citation biases, they remain invaluable tools for understanding broad research patterns and identifying emerging trends.

This analysis underscores the field’s transition toward a more integrated understanding of skin disease management, one that considers not only clinical efficacy but also patient experience, treatment accessibility, and long-term outcomes. As the discipline continues to evolve, this holistic approach to dermatological care promises to enhance treatment adherence and ultimately improve patient outcomes across diverse global populations.

## Figures and Tables

**Table 1:** Top 10 countries by scientific output and citation impact on adherence to treatments for chronic skin conditions (1975–2024).

Country	Papers on Chronic Skin Conditions	Citations	Average Citations/Document	Total Publications	Specialization %
USA	3615	121,411	33.59	14,974,321	0.00024
Germany	1237	49,034	39.64	3,451,029	0.00036
England	1136	47,249	41.59	3,949,154	0.00029
Italy	1052	30,447	28.94	2,245,001	0.00047
China	734	12,819	17.46	8,214,246	0.00009
Canada	679	29,343	43.22	2,463,580	0.00028
France	572	23,929	41.83	2,712,410	0.00021
India	563	7899	14.03	2,309,048	0.00024
Japan	558	18,834	33.75	3,249,993	0.00017
Spain	545	17,188	31.54	1,826,042	0.00030

**Table 2:** The 10 most significant publications in therapeutic adherence studies for chronic dermatological disorders (1975–2024).

Paper/Source	TC	TC/Year	Normalized TC	DOI
Finlay AY, 1994 CLIN EXP DERMATOL	3838	123.81	21.78	10.1111/j.1365-2230.1994.tb01167.x
Parisi R, 2013, J INVEST DERMATOL	1796	149.67	41.07	10.1038/jid.2012.339
Leonardi CL, 2008, LANCET	1311	77.12	23.70	10.1016/S0140-6736(08)60725-4
Armstrong AW, 2020, JAMA-J AM MED ASSOC	1177	235.40	57.76	10.1001/jama.2020.4006
Hay RJ, 2014, J INVEST DERMATOL	964	87.64	28.31	10.1038/jid.2013.446
Eichenfield LF, 2014, J AM ACAD DERMATOL	906	82.36	26.61	10.1016/j.jaad.2014.03.023
di Cesare A, 2009, J INVEST DERMATOL	861	53.81	18.55	10.1038/jid.2009.59
Krueger G, 2001, ARCH DERMATOL	814	33.92	12.30	NA
Williams HC, 2012, LANCET	790	60.77	21.20	10.1016/S0140-6736(11)60321-8
Weidinger S, 2018, NAT REV DIS PRIMERS	776	110.86	30.37	10.1038/s41572-018-0001-z

TC, total number of citations; Normalized TC, the total citations of a document adjusted by the average citations of all documents within the same field or year.

## Data Availability

The raw data supporting the conclusions of this article will be made available by the authors on request.

## Supplementary Materials

The following supporting information can be downloaded at: https://www.mdpi.com/article/10.3390/bioengineering12080890/s1, Supplementary File S1, Python script thesaurus generator.

## Abbreviations

The following abbreviations are used in this manuscript:

QoL	Quality of life
DLQI	Dermatology Life Quality Index
WOS	Web of Science
