# Supplementary material for: Mapping Emerging Scientific Trends in Chronic Skin Disorders Using Machine Learning-Based Bibliometrics
Source: Bioengineering (Basel). 2025 Aug 20;12(8):890. doi: 10.3390/bioengineering12080890 (PMC12383886; doi:10.3390/bioengineering12080890)
Supplement: Supplementary file 1 [file bioengineering-12-00890-s001.zip › bioengineering-3795372-supplementary.pdf]

thesaurus_generator.py

```
import pandas as pd
import numpy as np
from sklearn.feature_extraction.text import TfidfVectorizer
from sklearn.metrics.pairwise import cosine_similarity
from nltk.stem import PorterStemmer
from nltk.corpus import wordnet
import nltk
import re

class ThesaurusGenerator:
    def __init__(self, similarity_threshold=0.75):
        self.similarity_threshold = similarity_threshold
        self.ps = PorterStemmer()

        # Download required NLTK data
        try:
            nltk.download('wordnet', quiet=True)
            nltk.download('punkt', quiet=True)
            nltk.download('averaged_perceptron_tagger', quiet=True)
        except:
            pass

    def preprocess_terms(self, terms):
        """Clean and standardize terms"""
        processed_terms = []
        for term in terms:
            if isinstance(term, str) and term.strip():  # Check if term is a non-empty stri
ng
                # Convert to lowercase and remove special characters except hyphen
                term = re.sub(r'[^a-zA-Z\s-]', '', term.lower())
                # Remove extra whitespace
                term = ' '.join(term.split())
                processed_terms.append(term)
        return processed_terms

    def find_similar_terms(self, terms):
        """Find similar terms using TF-IDF and cosine similarity"""
        if not terms:
            print("Warning: No terms provided to process")
            return []

        # Preprocess terms
        processed_terms = self.preprocess_terms(terms)
        if not processed_terms:
            print("Warning: No terms remained after preprocessing")
            return []

        # Create TF-IDF vectors
        vectorizer = TfidfVectorizer(analyzer='char', ngram_range=(2, 3))
        tfidf_matrix = vectorizer.fit_transform(processed_terms)

        # Calculate cosine similarity
        similarity_matrix = cosine_similarity(tfidf_matrix)

        # Find groups of similar terms
        similar_groups = []
        used_terms = set()

        for i in range(len(terms)):
            if i not in used_terms:
```

```
                group = {i}
                used_terms.add(i)

                # Find similar terms
                for j in range(i + 1, len(terms)):
                    if j not in used_terms and similarity_matrix[i][j] > self.similarity_th
reshold:
                        group.add(j)
                        used_terms.add(j)

                if len(group) > 1:
                    similar_groups.append([terms[idx] for idx in group])

        return similar_groups

    def generate_thesaurus_file(self, keywords_string, output_file):
        """Generate VOSviewer thesaurus file from a comma-separated string of keywords"""
        # Split the keywords string into a list
        keywords = [k.strip() for k in keywords_string.split(',') if k.strip()]
        print(f"Processing {len(keywords)} keywords...")

        # Find similar terms
        similar_groups = self.find_similar_terms(keywords)
        print(f"Found {len(similar_groups)} groups of similar terms")

        # Generate thesaurus content
        thesaurus_content = []

        for group in similar_groups:
            # Use the shortest term as the preferred term
            preferred_term = min(group, key=len)
            for term in group:
                if term != preferred_term:
                    thesaurus_content.append(f"{term},{preferred_term}")

        # Sort entries alphabetically
        thesaurus_content.sort()

        # Write to file
        with open(output_file, 'w', encoding='utf-8') as f:
            for line in thesaurus_content:
                f.write(line + '\n')

        return len(thesaurus_content)

def main():
    with open('test.txt', 'r') as file:
    # Read the file and remove any extra spaces or newlines
        keywords = file.read().replace('\n', ',').replace('\r', '').strip(',')

    # Create the string in the desired format
    keywords_string = f'""" {keywords} """'
    # Your keywords string

    # Generate thesaurus file
    generator = ThesaurusGenerator(similarity_threshold=0.75)
    num_entries = generator.generate_thesaurus_file(keywords_string, 'vosviewer_thesaurus.t
xt')
    print(f"Generated thesaurus file with {num_entries} entries")
```

```
if __name__ == "__main__":
    main()
```
